# Evaluation of the Electronic Clinical Dementia Rating for Dementia Screening

**DOI:** 10.1001/jamanetworkopen.2023.33786

**Published:** 2023-09-14

**Authors:** Rachel L. Nosheny, Daniel Yen, Taylor Howell, Monica Camacho, Krista Moulder, Shilpa Gummadi, Chau Bui, Sandhya Kannan, Miriam T. Ashford, Kristen Knight, Connie Mayo, Maureen McMillan, Ronald C. Petersen, Nikki H. Stricker, Erik D. Roberson, Carol Chambless, Adam Gersteneker, Roy Martin, Richard Kennedy, Yue Zhang, Walter Kukull, Derek Flenniken, Juliet Fockler, Diana Truran, R. Scott Mackin, Michael W. Weiner, John C. Morris, Yan Li

**Affiliations:** 1 Department of Psychiatry and Behavioral Sciences, University of California, San Francisco; 2 Department of Radiology and Biomedical Imaging, University of California, San Francisco; 3 Northern California Institute for Research and Education, Department of Veterans Affairs Medical Center, San Francisco; 4 Knight Alzheimer’s Disease Research Center, Department of Neurology, Washington University School of Medicine, St Louis, Missouri; 5 Department of Neurology, Mayo Clinic, Rochester, Minnesota; 6 Division of Neurocognitive Disorders, Department of Psychiatry and Psychology, Mayo Clinic, Rochester, Minnesota; 7 Alzheimer’s Disease Center, Center for Neurodegeneration and Experimental Therapeutics, Department of Neurology, University of Alabama at Birmingham, Birmingham; 8 Division of Neuropsychology, Department of Neurology, The University of Alabama at Birmingham, Birmingham; 9 Division of Gerontology, Geriatrics, and Palliative Care, Department of Medicine, University of Alabama at Birmingham, Birmingham; 10 Department of Epidemiology, University of Washington, Seattle; 11 National Alzheimer’s Coordinating Center, Seattle, Washington; 12 Department of Biostatistics, Washington University School of Medicine, St Louis, Missouri

## Abstract

**Importance:**

The Clinical Dementia Rating (CDR) is a well-validated instrument widely used to detect and stage dementia due to Alzheimer disease. The digital Electronic Clinical Dementia Rating (eCDR) can be remotely self-administered and automatically scored, with potential to facilitate efficient dementia screening and staging.

**Objective:**

To evaluate the association of the eCDR with the CDR and other in-clinic assessments for screening older adults for cognitive impairment.

**Design, Setting, and Participants:**

This multisite, cross-sectional study used baseline data from a longitudinal, observational study from 2020 to 2023, including up to 3 years of follow-up. Participants were enrolled from 3 Alzheimer Disease Research Centers and the Brain Health Registry. Participants (aged ≥55 years, with a study partner, and no acute or unstable major medical conditions) were recruited during in-clinic visits or by automated emails.

**Exposures:**

Participants completed the Uniform Data Set Version 3 (UDS; including the CDR) in supervised clinical research settings, and then completed the eCDR remotely, online and unsupervised, using their own device.

**Main Outcomes and Measures:**

The primary outcomes were eCDR scores (item; categorical box and global; continuous box and global), CDR scores (item; categorical box and global), and UDS assessment scores. Associations were evaluated using linear and logistic regressions.

**Results:**

A total of 3565 participants were contacted, and 288 were enrolled. Among 173 participants with item-level data (mean [SD] age, 70.84 [7.65] years; 76 women [43.9%]), eCDR to CDR concordance was 90% or higher for 33 items (63%) and 70% to 89% for 13 items (25%). Box (domain) level concordance ranged from 80% (memory) to 99% (personal care). The global score concordance rate was 81%. κ statistics were fair to moderate. Among 206 participants with box and global scores (mean [SD] age, 71.34 [7.68] years; 95 women [46.1%]), eCDR continuous global score was associated with CDR global (categorical) score with an area under the receiver operating characteristic curve of 0.79 (95% CI, 0.70-0.87). Correlations between eCDR and in-clinic UDS assessments were similar to those between CDR sum of box scores and the same in-clinic assessments.

**Conclusions and Relevance:**

These findings suggest that the eCDR is valid and has potential use for screening and assessment of older adults for cognitive and functional decline related to Alzheimer disease. Instrument optimization and validation in diverse cohorts in remote settings are crucial for evaluating scalability and eCDR utility in clinical research, trials, and health care settings.

## Key Points

**Question:**

Is the digital, remotely administered Electronic Clinical Dementia Rating (eCDR) useful for dementia screening and assessment?

**Findings:**

In this cross-sectional study including 206 adults, the eCDR continuous global score was associated with the in-clinic CDR global categorical score (area under the curve, 0.79). For 173 adults with item-level data, concordance between the eCDR and the CDR for item, box, and global scores was moderate to high, with κ statistics in the fair to moderate range.

**Meaning:**

These findings suggest that the eCDR is valid and has potential for screening and assessing older adults for cognitive and functional decline related to Alzheimer disease.

## Teaser

This cross-sectional study evaluates the association of the Electronic Clinical Dementia Rating with the Clinical Dementia Rating and other in-clinic assessments for screening older adults for cognitive and functional decline related to Alzheimer disease.

## Introduction

Alzheimer disease (AD) is an immense and growing public health threat, causing cognitive and functional decline, disability, and death in millions of older adults.^1^ Billions of dollars are being invested to develop effective treatments.^2^ A major challenge is efficient identification of older adults at risk for and with cognitive impairment and dementia for recruitment, screening, assessment, and longitudinal monitoring in clinical research, clinical trials, and health care settings. Many mildly impaired people may not be seeking treatment. Accurate, reliable, and efficient strategies for screening and assessment are needed.^3,4,5,6,7^ Remote, digital assessments are a promising approach, with evidence accumulating of their feasibility and validity.^8,9,10,11,12^

The Clinical Dementia Rating (CDR) has been adopted worldwide as the standard benchmark for staging dementia. It assesses cognitive and functional decline across multiple, clinically relevant domains (ie, boxes): memory, orientation, judgment and problem solving, community affairs, home and hobbies, and personal care.^13^ It requires semistructured, assessor-conducted interviews with a participant and study partner. CDR attributes include face validity, linkage to validated diagnostic criteria for AD dementia,^14,15^ scoring that is independent of psychometric performance, lack of practice effects, and minimal influence of age, educational, and linguistic confounders.^16,17^ It exhibits high interrater reliability in multicenter trials,^18,19,20,21^ strong content and criterion validity,^22^ and internal consistency and internal responsiveness.^23^ The length of the interviews and the need for clinical judgment to score the CDR limit its scalability for screening and longitudinal monitoring.

The electronic Clinical Dementia Rating (eCDR) is a novel, online, digital instrument to monitor cognitive and functional change in older adults. The eCDR^24^ was developed on the basis of an item response theory (IRT) analysis of the CDR.^25^ The eCDR retains key properties of the CDR. It stages cognitive and functional impairment using separate responses from the participant and a study partner obtained from separate, online, unsupervised digital questionnaires. The eCDR can be self-administered on an individual’s own device, without the need for an assessor or any other assistance. It includes an automated scoring algorithm, based on IRT analysis, that generates both categorical (box and global) and novel continuous scoring outcomes.

The overall goal of this study was to evaluate the validity of the eCDR. We tested the hypotheses that (1) the eCDR and CDR demonstrate high item, box, and global concordance; (2) eCDR performance is associated with CDR score; and (3) correlations between eCDR scores and in-clinic neuropsychological test scores are similar to those between CDR scores and the same in-clinic tests.

## Methods

### Participants and Study Design

This cross-sectional analysis includes baseline data from an observational, longitudinal study and follows the Strengthening the Reporting of Observational Studies in Epidemiology (STROBE) reporting guidelines. Participants were recruited from (1) the University of California, San Francisco (UCSF) Brain Health Registry (BHR),^26^ and (2) National Institute on Aging Alzheimer Disease Research Centers (ADRCs) at Mayo Clinic (Rochester, Minnesota), University of Alabama at Birmingham, and Washington University (St Louis, Missouri). All participants were required to be aged 55 years or older, be fluent in English, have an available study partner, and have regular access and ability to use an internet-connected device. Exclusion criteria were self-report of an acute or uncontrolled major medical condition and recent history (<6 months) of abuse or dependence on drugs and/or alcohol. All study partners were required to be aged 18 years or older, be fluent in English, and have regular access and ability to use an internet-connected device. Study partners were required to have regular and frequent interaction (in person, by telephone, or online) with the participant, such that they could answer questions about the participant’s memory and day-to-day functioning. At UCSF, current BHR participants were referred to the study using automated email invitations with the following additional inclusion criteria: (1) previously agreed to be contacted about opportunities to participate in additional research and (2) resided within 50 miles of the clinic site. ADRC participants were recruited during regular clinical visits. Reasons for nonparticipation are summarized in eAppendix 1 in Supplement 1. All participants signed informed consent during their in-clinic visit, and an online informed consent document within BHR. All activities were performed under institutional review board approval at the local site and at UCSF.

### Electronic Clinical Dementia Rating

Participants and study partners completed the eCDR in-clinic at their baseline visit without assistance and then were instructed to complete the eCDR remotely, through the BHR online portal,^9^ within 2 weeks of the initial in-clinic administration (eAppendix 1 in Supplement 1). The mean (SD) time to complete the eCDR was 12.0 (3.4) minutes for participants and 16.0 (2.1) minutes for study partners.

### Demographic Variables

Participant characteristics of age, gender (self-reported as male, female, other, or prefer not to say), race, ethnicity, and years of education were collected through a remotely administered demographics questionnaire. Participants self-reported their race (African American, Asian, Native American, Pacific Islander, White, declined to state, or other) and their ethnicity (Latino or Hispanic, not Latino or Hispanic, or declined to state). For race categories, *other* is a distinct category that is a response option in the self-report race question. No additional race categories are included in *other*. Data on race and ethnicity were collected to assess the ethnocultural diversity of the sample, and so that contributions of race and ethnicity could be considered in future analyses.

### Uniform Data Set, Version 3

In-clinic Uniform Data Set Version 3, Initial Visit Packet (UDS) was collected for each participant at the ADRCs or at UCSF and uploaded to the National Alzheimer Coordinating Center. Then it was downloaded into the BHR database for linkage to eCDR data (eAppendix 1 in Supplement 1).

### Clinical Dementia Rating

CDR was obtained in-clinic as part of the UDS. Box scores were assigned according to clinical judgment across 3 levels of impairment: none (0), very mild (0.5), and mild (1). The overall (global) score was calculated from a standard algorithm.^13^ CDR sum of box (CDR-SB) scores were calculated as the sum of all box scores.^27^

### Statistical Analysis

#### Participant Characteristics

Continuous variables were summarized as mean (SD) and range (minimum to maximum). Categorical variables were summarized as count and percentage.

#### eCDR Scoring

eCDR scores were automatically generated using a scoring algorithm we previously developed on the basis of a bifactor IRT model with correlated domain-specific factors^25^ (eAppendix 1 in Supplement 1). The accuracies of eCDR IRT global score and domain-specific IRT scores (continuous) in classifying CDR global (0 or >0) and domain-specific CDR box scores (0 or >0) were evaluated using area under receiver operating characteristic curve (AUC) based on logistic regressions. The accuracies of eCDR IRT global score and domain-specific IRT scores (continuous) in association with CDR-SB scores were evaluated using linear regressions. A single eCDR scoring output was included in each model. Age, gender, and education were considered as covariates in the logistic and linear regressions. They were first assessed 1 covariate at a time, and then all together. Ten-fold cross-validation was performed for the logistic regressions and linear regression. Cross-validated AUCs and their 95% CIs and *R*^2^ were reported. The eCDR scoring algorithm was implemented using the R mirt package.^28^

#### Concordance Between CDR and eCDR

Concordance was defined as agreement between the CDR and eCDR for the item, box, or global score of each participant. Percentage concordance and weighted κ statistics for each item’s global score and each domain-specific box score were examined. Item-level eCDR data were available for all participants. Item-level CDR data were available for a subset of participants. The remaining participants enrolled from study sites that did not collect item-level CDR data and were, therefore, excluded from item-level concordance analyses. Statistical analyses were conducted with SAS statistical software version 9.4 (SAS Institute). All tests were 2-sided, and *P* < .05 was considered statistically significant.

## Results

### Participants

Of 3565 individuals contacted, 288 (8%) enrolled between March 2020 and April 2022; 251 (87%) had complete eCDR data, 45 of whom were excluded from analyses due to a delay between eCDR completion and linkage with in-clinic data. A total of 206 study participants (mean [SD] age, 71.34 [7.68] years; 95 women [46.1%]; mean [SD] years of education 17.15 [2.08]; 176 White [85.4%]) were included; 79% (163 participants) had CDR of 0, and 21% (43 participants) had CDR greater than or equal to 0.5 (Table 1). Participants had a mean (SD) CDR-SB of 0.22 (0.63) (median [IQR], 0 [0-0]). Eighty-four percent of participants (173 participants; mean [SD] age, 70.84 [7.65] age; 76 women [43.9%]; mean [SD] years of education, 17.12 [2.08]; 148 White [85.5%]) had an eCDR global score of 0, and 16% (33 participants) had an eCDR global score greater than or equal to 0.5 (eAppendix 2 and eTable 1 in Supplement 1). A subset of 173 participants had item-level eCDR and CDR data available for item-level concordance analyses. Among study partners, the completion rate was 90%. Enrollment rates were 41% from 3 ADRCs and 5% from the BHR.

**Table 1.** Participant Characteristics

Variable	Participants, No. (%)		
	Overall (n = 206)	CDR 0 (n = 163)	CDR ≥0.5 (n = 43)^a^
Age, mean (SD) [range], y	71.34 (7.68) [51 to 90]	70.91 (7.62) [51 to 90]	72.98 (7.81) [55 to 89]
Gender^b^			
Female	95 (46.1)	69 (42.3)	26 (60.5)
Male	111 (53.9)	94 (57.7)	17 (39.5)
Education, mean (SD) [range], y	17.15 (2.08) [12 to 20]	17.17 (2.03) [12 to 20]	17.09 (2.31) [12 to 20]
Race			
African American	10 (4.9)	7 (4.3)	3 (7.0)
Asian	17 (8.2)	14 (8.6)	3 (7.0)
White	176 (85.4)	139 (85.3)	37 (86.0)
Other^c^	3 (1.5)	3 (1.8)	0
Ethnicity			
Latino or Hispanic	9 (4.4)	6 (3.7)	3 (7.0)
Not Latino or Hispanic	197 (95.6)	157 (96.3)	40 (93.0)
CDR-SB category			
0	155	155	0
0.5	40^d^	8^e^	32^f^
1	5	0	5
1.5	1	0	1
2	1	0	1
3	1	0	1
4	1	0	1
4.5	1	0	1
5	1	0	1
CDR-SB overall, mean (SD) [range]	0.22 (0.63) [0 to 5]	0.02 (0.11) [0 to 0.5]	0.95 (1.10) [0.5 to 5]
eCDR global			
0	173 (84.0)	149 (91.4)	24 (55.8)
≥0.5	33 (16.0)	14 (8.6)	19 (44.2)
eCDR box score			
Community affairs domain			
0	194 (94.2)	160 (98.2)	34 (79.1)
≥0.5	12 (5.8)	3 (1.8)	9 (20.9)
Judgment and problem solving domain			
0	182 (88.4)	154 (94.5)	28 (65.1)
≥0.5	24 (11.6)	9 (5.5)	15 (34.9)
Memory domain			
0	168 (81.5)	146 (89.6)	22 (51.2)
≥0.5	38 (18.5)	17 (10.4)	21 (48.8)
Orientation domain			
0	180 (87.4)	154 (94.5)	26 60.5)
≥0.5	26 (12.6)	9 (5.5)	17 (39.5)
Personal care domain			
0	203 (98.5)	163 (100)	40 (93.0)
≥0.5	3 (1.5)	0	3 (7.0)
eCDR IRT score, mean (SD) [range]			
Global	−0.43 (0.34) [−0.84 to 0.81]	−0.51 (0.28) [−0.84 to 0.31]	−0.13 (0.38) [−0.84 to 0.81]
Community affairs domain	−0.46 (0.38) [−0.87 to 0.94]	−0.55 (0.31) [−0.87 to 0.71]	−0.14 (0.44) [−0.87 to 0.94]
Judgment and problem solving domain	−0.45 (0.31) [−0.83 to 0.58]	−0.52 (0.27) [−0.83 to 0.31]	−0.20 (0.33) [−0.83 to 0.58]
Memory domain	−0.45 (0.36) [−0.87 to 0.90]	−0.54 (0.29) [−0.87 to 0.29]	−0.14 (0.40) [−0.87 to 0.90]
Orientation domain	−0.41 (0.37) [−0.84 to 0.90]	−0.50 (0.31) [−0.84 to 0.34]	−0.09 (0.41) [−0.84 to 0.90]
Personal care domain	−0.33 (0.34) [−0.68 to 1.28]	−0.41 (0.26) [−0.68 to 0.42]	−0.01 (0.44) [−0.68 to 1.28]

Abbreviations: CDR, Clinical Dementia Rating; eCDR, Electronic Clinical Dementia Rating; IRT, item response theory; SB, sum of box.

^a^ Forty-one patients had a CDR of 0.5, and 2 patients had a CDR of 1.

^b^ Gender was self-reported as male, female, other, or prefer not to say. No patients reported other or prefer not to say.

^c^ For race categories, *Other* is a distinct category that is a response option in the self-report race question. No additional race categories are included in *Other*.

^d^ A total of 24.8% of participants had CDR-SB of 0.5 or higher.

^e^ A total of 5.0% of participants had CDR-SB of 0.5 or higher.

^f^ All participants had CDR-SB of 0.5 or higher.

### Concordance Between the Remote eCDR and In-Clinic CDR

**Figure 1.**
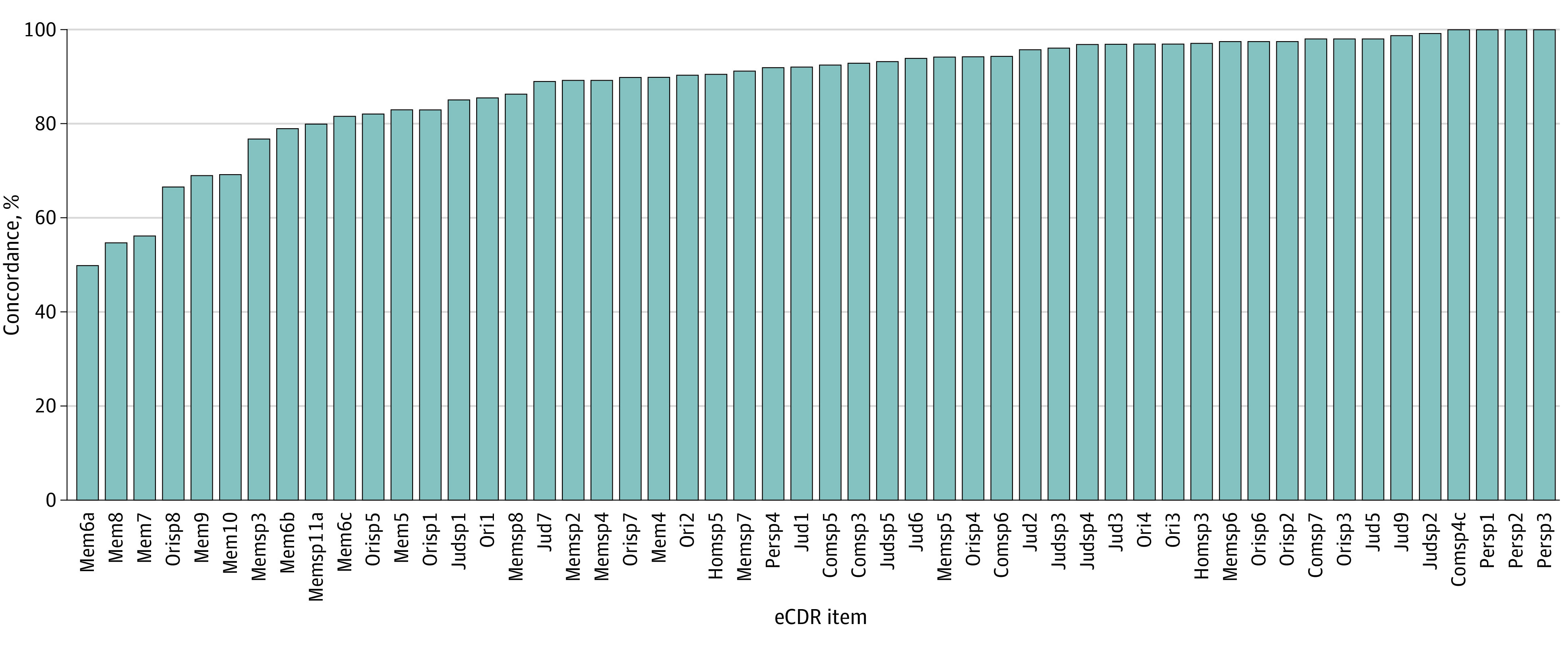
Item-Level Concordance Rate Between the Electronic Clinical Dementia Rating (eCDR) and Clinical Dementia Rating (CDR) For each eCDR item, the percentage concordance with the corresponding CDR items is shown. Com indicates community affairs box; hom, home and hobbies box; jud, judgment and problem solving box; mem, memory box; ori, orientation box; per, personal care box; and sp, study partner-report items. See eTable 2 in Supplement 1 for complete text of items.

Among 52 common items in the eCDR and the CDR, 33 items (63%) had 90% or higher concordance rate, 13 items (25%) had concordance rate between 70% and 89%, and 6 items (12%) had concordance less than 70% (Figure 1 and eTable 2 in Supplement 1). Box-level concordance ranged from 80% (memory) to 99% (personal Care) (Table 2). Concordance between eCDR and CDR global, categorical scores was 81%. κ statistics ranged from 0.31 (judgment and problem solving) to 0.54 (community affairs), with κ = 0.40 for global score (Table 2). A description of items with concordance less than 90% shows possible sources of discordance (eTable 3 in Supplement 1).

**Table 2.** Box and Global Concordance Between eCDR and CDR

Box	Concordance rate, %	κ (95% CI)	eCDR box score distribution			CDR box score distribution		
			0	0.5	1	0	0.5	1
Personal care	99	0.39 (−0.15 to 0.94)	203	0	3	204	0	2
Community affairs	96	0.54 (0.23 to 0.85)	194	11	1	202	3	1
Judgment and problem solving	88	0.31 (0.12 to 0.51)	182	24	0	193	12	1
Orientation	88	0.35 (0.15 to 0.55)	180	24	2	196	7	3
Memory	80	0.41 (0.26 to 0.56)	168	36	2	163	41	2
Global	81	0.40 (0.24 to 0.56)	173	32	1	163	41	2

Abbreviations: CDR, Clinical Dementia Rating; eCDR, Electronic Clinical Dementia Rating.

### Association of eCDR With CDR

**Figure 2.**
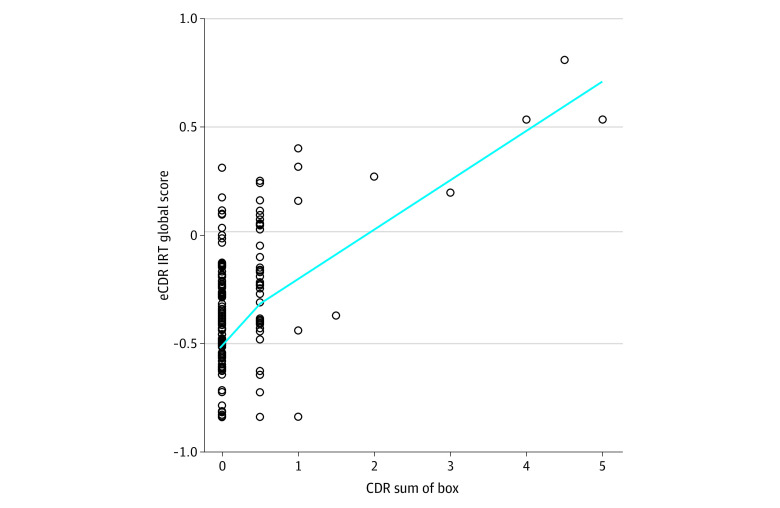
Association of Electronic Clinical Dementia Rating (eCDR) Continuous Item Response Theory (IRT) Global Score and Clinical Dementia Rating (CDR) Sum of Box Scatter plot shows eCDR IRT global scores and CDR sum of box scores (Spearman correlation, 0.43; 95% CI, 0.31-0.53).

The eCDR IRT global score was significantly correlated with CDR-SB score (*r*, 0.43; 95% CI, 0.31-0.53) (Figure 2). In linear models adjusting for age, gender, and education, the eCDR IRT global score was significantly associated with CDR-SB (β, 0.95; SE, 0.11; *P* < .001; *R*^2^, 0.27; cross-validated *R*^2^, 0.22).

### eCDR Classification of CDR Categories

**Figure 3.**
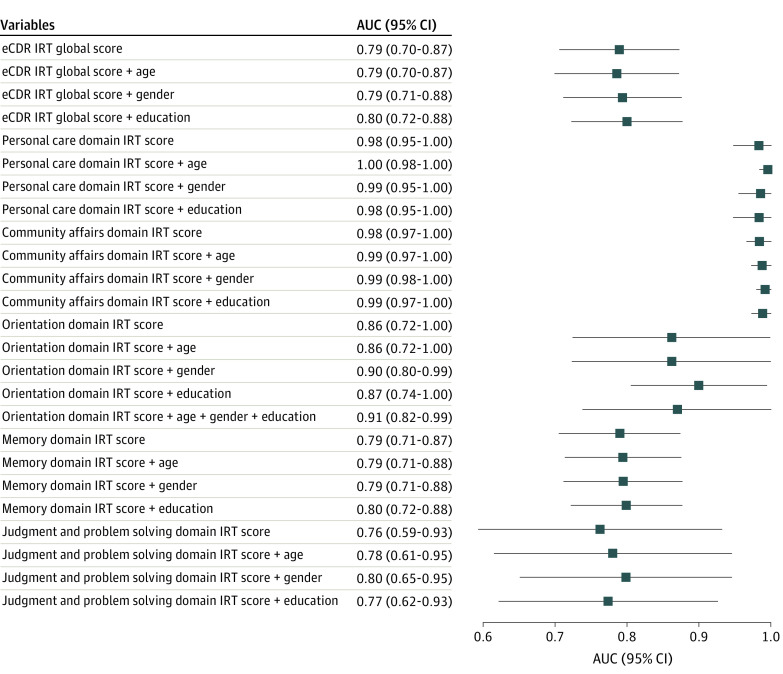
Forest Plot of the Area Under the Receiver Operating Characteristic Curve (AUC) for the Electronic Clinical Dementia Rating (eCDR) Scoring Outputs Association With CDR Global Score eCDR scoring outputs are listed in the left column. A single eCDR scoring output was used in each model. IRT indicates item response theory.

eCDR IRT scoring outputs (box and global) were associated with CDR categorical scores (box and global), with AUCs ranging from 0.76 to 0.98 (Figure 3 and eTable 4 in Supplement 1). The highest accuracy was for the personal care (AUC, 0.98; 95% CI, 0.95-1.00) and community affairs (AUC, 0.98; 95% CI, 0.97-1.00) boxes. The lowest accuracy was found for the memory (AUC, 0.79; 95% CI, 0.71-0.87) and judgment and problem solving (AUC, 0.76; 95% CI, 0.59-0.93) boxes. The AUC for the eCDR global score was 0.79 (95% CI, 0.70-0.87). Adjusting for age, gender, and education by adding them as covariates had a small effect on the accuracy of the models. Time interval between CDR and eCDR was not a significant variable in the models and did not change the AUCs or adjusted *R*^2^ when it was included as a covariate (eFigure and eTable 5 in Supplement 1). Of note, the eCDR global categorical score misclassified 24 individuals (55.8%) with CDR greater than 0 as unimpaired, and 8.6% of participants with CDR of 0 (14 participants) had false-positive eCDR findings.

## Discussion

This cross-sectional study found that the eCDR administered remotely had high concordance with the in-clinic CDR at the item, box, and global levels. Agreement beyond chance (ie, κ statistics) was in the fair to moderate range. The lowest concordance was for the memory box (80%) and the global score (81%). The study also found that eCDR scores correlated with CDR-SB scores and were associated with CDR scores with moderate to high accuracy. Finally, eCDR scores were correlated with UDS neuropsychological assessments at levels similar to the correlation between CDR scores and the same assessments. Overall, the results support the feasibility and validity of the novel eCDR and its potential to be used in different settings to efficiently identify older adults with cognitive and functional impairments relevant to AD. Crucial next steps are optimization of eCDR content and scoring, validation in diverse populations, and investigation of the ability of the eCDR to track longitudinal change in cognition and function in completely remote settings.

Our results support the feasibility and usability of the eCDR instrument administered remotely through a website, consisting of separate digital surveys for participants and study partners. Enrollment rates were 41% from 3 ADRCs and 5% from the BHR. These are in line with rates from comparable studies with similar burden. High compliance was demonstrated by completion rates of 87% (participants) and 90% (study partners). These rates are substantially higher than those of other BHR surveys.^9^ Still, the drop-off from 3565 study invitations to 288 individuals enrolled (8% of those invited) suggests that there may be study selection biases and emphasizes the need for caution regarding CDR scalability in other settings.

The eCDR retains key properties of the very widely used and well-validated CDR, which is a primary end point for multiple pharmacological intervention clinical trials.^29,30^ Like the CDR, the eCDR stages both cognitive and functional impairment and decline across multiple domains (ie, boxes). It mitigates cultural and educational biases by rating participants relevant to their own baselines, removing reliance on normative data, and incorporating indices of functional status. Compared with the CDR, the eCDR has added advantages, including the potential to lessen the burden of in-clinic research visits^31,32^ by using remote administration with automated scoring. This study is a first step in establishing the feasibility and validity of the eCDR in a sample of convenience. If the eCDR is further validated and optimized, it has potential applications for screening and longitudinal assessment of cognition and function to facilitate clinical research, clinical trials, and health care.

One measure of eCDR validity is its association with the in-clinic CDR. Our results demonstrate high overall concordance (agreement) between eCDR and CDR item, box, and global scores. When considering agreement beyond chance levels, κ statistics were in the fair-to-moderate range (0.31-0.54; κ = 0.40 for global score). For the eCDR categorical scores, the high level of overall concordance was related to agreement at the CDR of 0 level and was less optimal for individuals with CDR greater than 0. Using CDR global as the criterion standard, the eCDR global categorical score misclassified 55.8% of individuals with CDR greater than 0 as unimpaired. This misclassification decreases the efficiency of using the eCDR as a scalable, first-step screening tool to identify older adults with cognitive impairments remotely. eCDR false-positives (8.6% of participants with CDR of 0), on the other hand, have important implications for its use to identify suitable candidates to receive AD therapeutics indicated for those with cognitive impairment, especially considering the potential risks of such treatments. The eCDR misclassification of individuals with CDR greater than 0 highlights the need for eCDR content and scoring optimization and additional studies to address the utility of the eCDR for screening and assessment of older adults.

A novel component of the eCDR is the IRT-derived continuous scoring outcomes (in addition to categorical measures, like the CDR). eCDR continuous IRT scores identified those with CDR greater than 0 with moderate-to-high accuracy (AUC for eCDR global score, 0.79) (Figure 3 and eTable 4 in Supplement 1). eCDR continuous IRT scores were also significantly associated with CDR-SB scores (Figure 2). These results demonstrate the potential of the eCDR to identify subtle cognitive and functional decline early in the AD continuum, including in preclinical (unimpaired and biomarker positive) and prodromal (early symptomatic and biomarker positive) AD. For example, individuals who have impaired scores on multiple CDR items can still be assigned a CDR of 0 and CDR-SB of 0, but their eCDR scores are greater than 0. Because detecting decline and impairment at these stages is a critical challenge in the field, our results support the use of the eCDR continuous IRT score in future studies.

Possible sources of the CDR to eCDR discordance can be identified by considering the analysis of item-level data, in which 12% of common items between the CDR and eCDR have concordance less than 70%. Many of these items include altered response options in the eCDR vs the CDR (eg, multiple choice vs open-ended response), a requirement for matching participant and study partner responses to assess dyad agreement (eg, “What was the last school you/the participant attended?”), and/or free-text responses (eTable 3 in Supplement 1). The 80% overall concordance in the memory box may also be partly due to the omission of the CDR autobiographical recall question,^33^ in which participants are asked about details of recent events already described by the study partner. This question, which was found to be associated with overall CDR score in our past IRT analysis,^25^ was eliminated owing to the difficulty of automatically assessing agreement of open-ended responses between the participant and study partner.

To address these issues, we plan to optimize the eCDR instrument. The eCDR scoring algorithm was developed on the basis of an IRT analysis of the CDR in 2894 cases.^25^ Thus, the eCDR scoring algorithm is currently optimized for CDR content, not eCDR content. We plan to conduct an IRT analysis of eCDR data, similar to the previous CDR IRT analysis. From best-fitting bifactor IRT models,^34^ we will compute weights of individual eCDR items, which will be incorporated into the continuous box and global scoring algorithms. We will also consider additional eCDR optimization approaches. First, we plan to implement improved methods to automatically match free-text responses between the participant and study partner to assess dyad agreement. Second, we will consider incorporating additional eCDR content into the scoring algorithm to address the absence of assessor or clinician judgment in assigning box scores. Supplemental content includes dyad familiarity (eg, time spent together, years known, and type of relationship) and subjective memory decline details (eg, date of onset, consistency, and frequency). These questions are already asked in the eCDR but are not part of the current scoring algorithm. Their incorporation into the scoring algorithm may help account for the reliability of the study partner and the clinical meaningfulness of reported subjective cognitive and functional decline. Third, both the in-clinic CDR and eCDR require an available study partner. This constraint limits accessibility, especially in those from historically underincluded populations.^35,36,37^ The eCDR further requires the participant to have sufficient device and internet access and the ability to navigate through the website and digital survey. This requirement may be a barrier for some older adults with cognitive impairment. Therefore, we will develop separate eCDR scoring algorithms that rely solely on participant or study partner information.

Although the association of the CDR with the eCDR is important for evaluating the utility of the eCDR, it is also possible that some features of the eCDR make it a more accurate reflection of an individual’s underlying cognitive and functional status than the CDR. The CDR has previously been found to have high interrater reliability.^25^ However, the CDR requirement for assessor clinical judgment to assign the box scores can introduce variability. The eCDR does not require assessor clinical judgment and instead relies on an automated scoring algorithm. Because the eCDR is completed in an unsupervised setting, it is possible that individuals may be more likely to provide candid responses, since they may not be intimidated by the presence of the assessor or the assessor learning about their cognitive impairment. Although eCDR content is modeled closely on CDR content, there are important differences. This raises the possibility that the eCDR is measuring different underlying constructs. Our analyses examining the association of the eCDR or CDR with numerous UDS assessments show similar correlation levels, with differences that are not clinically meaningful. In future studies, we plan to further investigate the association of the eCDR with additional outcomes relevant to AD and related dementia, including (1) the correlation between eCDR box scores and domain-specific cognitive assessments, (2) associations between eCDR and clinical progression, and (3) the association of the eCDR with AD biomarkers.

### Limitations

Multiple cohort characteristics limit the generalizability of the study results. These include lack of ethnocultural, language, and educational diversity; selection biases for older adults with adequate device and internet access and literacy to complete unsupervised digital assessments; an available study partner; previous engagement in AD research; and previous exposure to the CDR. The eCDR can be used independently only by individuals who can navigate through the website and the digital surveys. However, internet use by older adults is increasing.^38,39^ The next generation at risk for cognitive decline, mild cognitive impairment, and AD is highly likely to have much greater technology access and literacy. The usability and validity of the eCDR in individuals with moderate and severe dementia (CDR >1) is not known because they were excluded from the current study. Only 21% of study participants had CDR greater than 0. Validation in diverse populations with a higher percentage of impaired participants and in settings outside of ADRCs and BHR are crucial next steps. Although we evaluated performance on a version of the eCDR obtained remotely and unsupervised, all participants previously had taken an in-clinic version of the eCDR. Further studies are needed to confirm the findings in completely remote settings. Owing to logistical constraints of the study, all participants completed the CDR before the eCDR. Thus, possible effects of order of administration could not be investigated.

## Conclusions

These findings suggest that the eCDR is a novel digital assessment with the potential to facilitate AD and related dementia research and health care by efficiently screening and assessing older adults. Further evaluation, validation, and optimization of the eCDR are needed.

## Notes

Supplement 1. **eAppendix 1.** Supplemental Methods **eAppendix 2.** Supplemental Results **eTable 1.** Participant Characteristics for Participants With Available Item-Level Clinical Dementia Rating (CDR) Data **eTable 2.** Complete Text of eCDR Questions Included in the Item-Level Analysis **eTable 3.** Characteristics of eCDR Items With CDR Concordance <90% **eTable 4.** Classification Accuracy of eCDR Association With CDR **eFigure.** Heat Map of Correlations Between CDR or eCDR and UDS Neuropsychological Assessments **eTable 5.** Difference in Correlations With Uniform Data Set (UDS) Assessments Between eCDR IRT Score and CDR-Sum of Boxes
eReferences

Supplement 2.
Data Sharing Statement
