# Supplementary material for: Evaluation of the Electronic Clinical Dementia Rating for Dementia Screening
Source: JAMA Netw Open. 2023 Sep 14;6(9):e2333786. doi: 10.1001/jamanetworkopen.2023.33786 (PMC10502518; doi:10.1001/jamanetworkopen.2023.33786)
Supplement: Supplement 1. — eAppendix 1. Supplemental Methods eAppendix 2. Supplemental Results eTable 1. Participant Characteristics for Participants With Available Item-Level Clinical Dementia Rating (CDR) Data eTable 2. Complete Text of eCDR Questions Included in the Item-Level Analysis eTable 3. Characteristics of eCDR Items With CDR Concordance <90% eTable 4. Classification Accuracy of eCDR Association With CDR eFigure. Heat Map of Correlations Between CDR or eCDR and UDS Neuropsychological Assessments eTable 5. Difference in Correlations With Uniform Data Set (UDS) Assessments Between eCDR IRT Score and CDR-Sum of Boxes eReferences [file jamanetwopen-e2333786-s001.pdf]

Supplemental Online Content

Nosheny RL, Yen D, Howell T, et al. Evaluation of the Electronic Clinical Dementia Rating for dementia screening. *JAMA Netw Open*. 2023;6(9):e2333786. doi:10.1001/jamanetworkopen.2023.33786

eAppendix 1. Supplemental Methods
eAppendix 2. Supplemental Results
eTable 1. Participant Characteristics for Participants With Available Item-Level Clinical Dementia Rating (CDR) Data
eTable 2. Complete Text of eCDR Questions Included in the Item-Level Analysis
eTable 3. Characteristics of eCDR Items With CDR Concordance <90%
eTable 4. Classification Accuracy of eCDR Association With CDR
eFigure. Heat Map of Correlations Between CDR or eCDR and UDS Neuropsychological Assessments
eTable 5. Difference in Correlations With Uniform Data Set (UDS) Assessments Between eCDR IRT Score and CDR-Sum of Boxes
eReferences

This supplemental material has been provided by the authors to give readers additional information about their work.

© 2023 Nosheny RL et al. *JAMA Network Open.*

eAppendix 1. Supplemental Methods

S1.1. Reasons for non-participation in the study. Reasons for non-participation in the study were recorded for 179 participants. Study staff could indicate multiple decline reasons for each potential participant. Reasons included overall lack of interest (n=74), other reason (n=42), time burden (n=24), inability to use a computer (n=17), study partner did not want to participate (n=14), medical exclusions (n=13), no reason (n=7), lack of access to a computer or internet at home (n=2), technical difficulties (n=1), deceased (n=1), staff decided participant should not participate (n=1).

S1.2. eCDR administration. The eCDR consists of separate Qualtrics surveys for the participant and study partner. To access the eCDR, participants and study partners each signed in to the online Brain Health Registry (BHR) portal separately using a username and password, and were presented with a series of online tasks. The BHR online portal, and related Study Partner Portal, have previously been used to enroll and assess >100,000 participants and study partners since 2014 [1,2]. The eCDR was self-administered on individuals' own Internet-connected devices, without any assistance from an assessor or other professional. Participants and study partners each complete their portion of the eCDR independently from each other. Written instructions were to complete the eCDR without assistance or input from each other or anyone else. Participants and study partners then received email reminders to complete the eCDR at 6-month intervals. The median time interval between the in-clinic eCDR and remote eCDR administration was 7 days (interquartile range= 4 - 13 days; range = 0 - 357 days; n=7 with time interval > 6 months). Analyses measuring agreement between eCDR global scores in-clinic versus remote indicated minimal effects of administration setting (Odds Ratio=0.99; 95% confidence interval: 0.64-1.53, p=1). Analyses reported herein include only the remote eCDR administration.

S1.3. eCDR scoring algorithm. The eCDR was scored automatically using a scoring algorithm we previously developed based on a bi-factor Item Response Theory (IRT) model with correlated domain-specific factors, yielding continuous and categorical box-specific and overall eCDR scores [3]. Briefly, based on 53 selected items and the Clinical Dementia Rating (CDR) latent structure, we compared the fit of four predefined models and selected the best fitting bi-factor IRT model with correlated domain scores. From this model we extracted two scores: (1) domain (box) scores: estimated as a weighted composite of the general factor and the corresponding domain specific factor; and (2) the overall (global) score: estimated as a weighted composite of

© 2023 Nosheny RL et al. *JAMA Network Open.*

the general factor and all the domain specific factors. The item discrimination parameters for the general factor and the domain specific factors were used to compute the weights according to the Bi-factor M4 method[4]. The continuous version of the eCDR scores (eCDR IRT scores) were derived from the bi-factor IRT model first, which represent the degree of impairment globally (IRT global score) and in each domain (domain specific IRT score). Categorical eCDR global and domain specific box scores were then generated by applying the optimal cutoffs previously developed [3]. For both continuous and categorical eCDR scores, higher values represent greater levels of impairment. The Home and Hobbies domain was not included in the scoring algorithm since the CDR item level dataset originally used to develop the scoring algorithm only had one item with data available [3].

S1.4. Uniform Data Set, Version 3. (UDS). UDS assessments[5-14] included: CDR, Montreal Cognitive Assessment (MoCA; total score, uncorrected for education), Craft Story 21 Learning (total learning, story units), Craft Story Delayed Recall (score) Benson Complex Figure Copy (copy score, total delayed recall score), Number Span Test (number correct trials, forward and backward), Trail Making A and B (completion time), Multilingual Naming (total score without semantic cue), Verbal Fluency (total correct), Functional Activities Questionnaire (FAQ; total score).

S1.5. Statistical Analyses

S1.5.1. Associations with UDS assessments. Association among CDR-SB, eCDR IRT score and other UDS neuropsychological assessments were examined using Spearman correlation and visualized using heatmap.

S1.5.2. Sensitivity analyses. Sensitivity analyses were conducted on a subset of participants who completed the CDR and eCDR within 14 days of each other (n=160).

© 2023 Nosheny RL et al. *JAMA Network Open.*

eAppendix 2. Supplemental Results

S2.1. Correlation with UDS assessments. Correlation between eCDR global IRT score and UDS neuropsychological test scores ranged from r=0.05 (MINT naming test) to r=0.349 (Trail Making Test B) (**eFigure**). For all UDS assessments, correlations between UDS assessment and eCDR IRT global score were not significantly different than correlations between UDS assessment and CDR-SB score (**eTable 5**).

S2.2. Sensitivity analyses. Sensitivity analyses conducted on a subset of participants who completed the CDR and eCDR within 14 days of each other (n=160) produced similar results for eCDR to CDR concordance, eCDR association with CDR, and eCDR correlation with UDS assessments.

© 2023 Nosheny RL et al. *JAMA Network Open.*

eTable 1: Participant Characteristics for Participants With Available Item-Level Clinical Dementia Rating (CDR) Data

Variable	Overall, N=173	CDR 0, N=133	CDR ≥ 0.5, N=40 (0.5, n=38; 1, n=2)
Age, y	70.84 ± 7.65 (55 - 89)	70.02 ± 7.34 (55 - 84)	73.58 ± 8.08 (55 - 89)
Gender (% Female)	76 (43.9%)	51 (38.4%)	25 (63.0%)
Education	17.12 ± 2.08 (12 - 20)	17.14 ± 2.00 (12 - 20)	17.05 ± 2.33 (12 - 20)
Race			
African America	7 (4.1%)	4 (3%)	3 (7.5%)
Asian	15 (8.7%)	14 (10.5%)	1 (2.5%)
White	148 (85.5%)	112 (84.2%)	36 (90.0%)
Other	3 (1.7%)	3 (2.3%)	0 (0%)
Ethnicity			
Latino/Hispanic	9 (5.2%)	6 (4.5%)	3 (7.5%)
Not Latino/Hispanic	164 (94.8%)	127 (95.5%)	37 (92.5%)
CDR SB			
0/0.5/1/1.5/2/4/4.5/5 (% ≥0.5)	127/36/5/1/1/1/1/1 (26.6%)	127/6/0/0/0/0/0/0 (4.5%)	0/30/5/1/1/1/1/1 (100%)
CDR SB	0.23 ± 0.65 (0 - 5)	0.02 ± 0.10 (0 - 0.5)	0.93 ± 1.08 (0.5 - 5)
eCDR global			
0	148 (85.5%)	125 (94.0%)	23 (57.5%)
≥0.5	25 (14.5%)	8 (6.0%)	17 (42.5%)
eCDR box score			
Community Affairs Domain			
0	165 (95.4%)	132 (99.2%)	33 (82.5%)
≥0.5	8 (4.6%)	1 (0.8%)	7 (17.5%)
Judgment and Problem Solving Domain			
0	156 (90.2%)	128 (96.2%)	28 (70.0%)
≥0.5	17 (9.8%)	5 (3.8%)	12 (30.0%)
Memory Domain			
0	142 (82.1%)	121 (91.0%)	21 (52.5%)
≥0.5	31 (17.9%)	12 (9.0%)	19 (47.5%)
Orientation Domain			
0	153 (88.4%)	128 (96.2%)	25 (62.5%)
≥0.5	20 (11.6%)	5 (3.8%)	15 (37.5%)
Personal Care Domain			
0	171 (98.8%)	133 (100%)	38 (95.0%)
≥0.5	2 (1.2%)	0 (0%)	2 (5.0%)
eCDR IRT score			
Global	-0.44 ± 0.34 (-0.84 - 0.81)	-0.53 ± 0.27 (-0.84 - 0.18)	-0.13 ± 0.37 (-0.84 - 0.81)
Community Affairs Domain	-0.47 ± 0.37 (-0.87 - 0.94)	-0.57 ± 0.30 (-0.87 - 0.71)	-0.14 ± 0.42 (-0.87 - 0.94)

© 2023 Nosheny RL et al. *JAMA Network Open.*

Judgment and Problem Solving Domain	-0.46 ± 0.31 (-0.83 - 0.58)	-0.54 ± 0.26 (-0.83 - 0.31)	-0.21 ± 0.31 (-0.83 - 0.58)
Memory Domain	-0.45 ± 0.35 (-0.87 - 0.90)	-0.55 ± 0.28 (-0.87 - 0.26)	-0.13 ± 0.40 (-0.87 - 0.90)
Orientation Domain	-0.41 ± 0.37 (-0.84 - 0.90)	-0.51 ± 0.29 (-0.84 - 0.23)	-0.08 ± 0.42 (-0.84 - 0.90)
Personal Care Domain	-0.34 ± 0.33 (-0.68 - 1.28)	-0.43 ± 0.25 (-0.68 - 0.42)	-0.04 ± 0.40 (-0.68 - 1.28)

CDR=Clinical Dementia Rating; SB=Sum of Boxes; eCDR=Electronic Clinical Dementia Rating; IRT=Item Response Theory. Continuous variables were summarized mean ± SD, (min - max). Categorical variables were summarized as count and percentage. For race categories, “Other” is a distinct category that is a response option in the self-report race question.

© 2023 Nosheny RL et al. *JAMA Network Open.*

eTable 2: Complete Text of eCDR Questions Included in the Item-Level Analysis

Item code	Question text
mem6a	What was the name of the last school you attended?
mem8	What is the field of your MOST RECENT major occupation/job (or your spouse's most recent major occupation/job, if not employed)?
mem7	What was your main occupation job (or spouse if not employed)?
orisp8	How often can he/she find his/her way about indoors?
mem9	When did you (or your spouse) retire and why?
mem10	Repeat the name and address I asked you to remember
memsp3	Can he/she remember a short list of items (shopping)?
mem6b	Where was the last school you attended? (Place of last school)
memsp11a	Where was he/she born?
mem6c	What grade did you complete at the last school you attended?
orisp5	Does he/she have difficulty with time relationships (when events happened in relation to each other)?
mem5	Where were you born?
orisp1	How often does he/she know of the exact: date of the month?
ori1	What is the date today?
judsp1	In general, if you had to rate his/her abilities to solve problems at the present time, would you consider the: as good as they have ever been; good, but not as good as before; fair; poor; no ability at all
memsp8	Does he/she completely forget important information from the distant past (e.g., birthdate, wedding date, place of employment)?
jud7	Subtract 3 from 20 and keep subtracting 3 from each new number all the way down.
memsp2	Can he/she recall recent events?
memsp4	Has there been some decline in memory during the past year?
orisp7	How often does he/she know how to get from one place to another outside of his/her neighborhood? (Usually; sometimes; rarely; don’t know)
mem4	When were you born?
ori2	What day of the week is it?
homsp5	Is he/she able to perform household chores at the level of (pick one. Informant does not need to be asked directly): No meaningful function (performs simple activities, such as making a bed, only with much supervision); functions in limited activities only (with some supervision, washes dishes with acceptable cleanliness; sets table); functions independently in some activities (operates appliances, such as a vacuum cleaner; prepares simple meals); functions in usual activities but not at usual level; normal function in usual activities.
memsp7	Does he/she forget pertinent details of the major event?
persp4	What is your estimate of his/her mental ability in the following areas: sphincter control (blessed) [normal complete control; occasionally wets bed; frequently wets bed; doubly incontinent]
jud1	How are these things alike? … turnip..cauliflower
comsp3	Does the subject have significant difficulty in his/her job because of problems with memory or thinking?
judsp5	Can he/she understand situations or explanations? (usually; sometimes; rarely; don’t know)
comsp5	If he/she is still driving, are there problems or risks because of poor thinking?
jud6	How many quarters in $6.75?
memsp5	Is his/her memory impaired to such a degree that it would have interfered with his/her activities of daily life a few years ago (or pre-retirement activities)? (Collateral sources opinion)
orisp4	How often does he/she know the exact day of the week?
comsp6	Is he/she able to independently shop for needs? (Rarely or never; sometimes; usually; don’t know)
jud2	How are these things alike? …desk…bookcase
judsp3	Rate his/her ability to handle complicated financial or business transactions (e.g., balance a checkbook, pay bills): (no loss; some loss; severe loss)
judsp4	Can he/she handle a household emergency (e.g., plumbing leak; small fire)? (as well as before; worse than before because of trouble thinking; worse than before, another reason)
jud3	What is the difference between these things?...lie…mistake

© 2023 Nosheny RL et al. *JAMA Network Open.*

ori4	What is the year?
ori3	What is the month?
homsp3	If in nursing home, what can he/she no longer do well (Home and Hobbies)?
memsp6	Does he/she completely forget a major event (e.g., trip, party, family wedding) within a few weeks of the event?
orisp6	Can he/she find his/her way about familiar streets?
orisp2	How often does he/she know the exact month?
comsp7	Is he/she able to independently carry out activities outside the home? (Rarely or never; sometimes; usually; don't know)
orisp3	How often does he/she know the exact year?
jud5	How many nickels in a dollar?
jud9	Subject's assessment of disability and station in life understanding of why she/he is present at the examination (may have covered, but rate here): good insight; partial insight; little insight
judsp2	Rate his/her ability to cope with small sums of money (e.g., make change, leave a small tip) :(no loss; some loss; severe loss)
comsp4c	If the subject no longer drives a car, is this because of memory or thinking problems?
persp1	What is your estimate of his/her mental ability in the following areas: dressing (blessed) [unaided; occasionally misplaced buttons, etc.; wrong sequence commonly forgotten items; unable to dress]
persp2	What is your estimate of his/her mental ability in the following areas: washing, grooming [unaided; needs prompting; sometimes needs help; always or nearly always needs help]
persp3	What is your estimate of his/her mental ability in the following areas: eating habits [clean, proper utensils; messily, spoon; simple solids; has to be fed completely)

eTable 3: Characteristics of eCDR Items With CDR Concordance <90%

eCDR Question	% concor-dance	Box	Respon-dent	Requires dyad agree-ment	Free text	Altered response format vs. CDR	CDR more impaired	eCDR more impaired
What was the last school you attended [name]?	50%	Mem	P	Yes	Yes	Yes	1%	49%
What was your last major job (or spouse if not employed)?	55%	Mem	P	Yes	No	Yes	5%	41%
What was your main occupation job (or spouse if not employed)? [2 field, position]	56%	Mem	P	Yes	No	Yes	6%	38%
How often can <name> find their way about indoors?	67%	Ori	SP	No	No	No	0%	33%
When did you retire and why?	69%	Mem	P	Yes	No	No	14%	17%
Repeat the name and address I asked you to remember [John Brown 42 Market Street]	69%	Mem	P	No	Yes	Yes	11%	20%
Can < name> remember a short list of 5 items without written reminders?	77%	Mem	SP	No	No	No	4%	19%
What was the last school you attended [Place]?	79%	Mem	P	Yes	Yes	Yes	14%	7%
Does < name> have a problem with his/her memory or thinking?	80%	Mem	SP	No	No	No	16%	4%
What was the last school you attended [grade]?	82%	Mem	P	Yes	No	Yes	8%	11%
Does < name> have difficulty with time relationships?	82%	Ori	SP	No	No	No	6%	11%
Where were you born [City and State]?	83%	Mem	P	Yes	No	Yes	12%	5%
How often does <name> know of the exact: Date of the Month?	83%	Ori	SP	No	No	No	12%	5%
In general, if you had to rate his/her abilities to solve problems at the present time, would you consider them: As good; Good, but not as good as before; Fair; Poor; No ability at all	85%	Jud	SP	No	No	No	7%	8%
What is the date today? [3 data elements]	86%	Ori	P	No	No	Yes	4%	10%
Does < name> forget important information of the distant past (10 or more years ago)? Examples are date of birth, wedding date, place of employment.	86%	Mem	SP	No	No	No	4%	10%
Subtract 3 from 20 and keep subtracting 3 from each new number all the way down [6 data elements]	89%	Jud	P	No	Yes	Yes	4%	7%
Can <name> recall recent events in which he/she participated?	89%	Mem	SP	No	No	No	5%	5%

© 2023 Nosheny RL et al. *JAMA Network Open.*

Can <name> recall recent events?	89%	Mem	SP	No	No	No	5%	5%
Has there been some decline in memory over the past year?	89%	Memory	SP	No	No	No	8%	3%

Mem=memory box**; Ori**=Orientation box**; Jud**=Judgment/Problem Solving Box
P=Participant; **SP**=Study Partner
Requires Dyad Agreement: Scoring of item requires comparison of the responses from the participant and study partner. If the participant's response matches the study partner's response, the item is scored as correct.
Free text: the response is an open text field
Altered response format vs CDR: The response options differ from the CDR (e.g, multiple choice or drop-down menus)
CDR more impaired: The percentage of instances in which the CDR scored the individual at a greater level of impairment than the eCDR
eCDR more impaired: The percentage of instances in which the eCDR scored the individual at a greater level of impairment than the CDR

© 2023 Nosheny RL et al. *JAMA Network Open.*

eTable 4: Classification Accuracy of eCDR Association With CDR

Outcome	Independent Variable(s)	AUC (95%CI)	p-value	cvAUC* (95%CI)
CDR Personal Care Domain box score	eCDR Personal Care Domain IRT score	0.98 (0.95-1.00)	<.0001	
	eCDR Personal Care Domain IRT score + age	1.00	<.0001	
	eCDR Personal Care Domain IRT score + gender	0.99 (0.95-1.00)	<.0001	
	eCDR Personal Care Domain IRT score + education	0.98 (0.95-1.00)	<.0001	
	eCDR Personal Care Domain IRT score + age + gender + education	1.00	<.0001	
	age + gender + education	0.94 (0.84-1.00)	<.0001	
CDR Community Affairs Domain box score	eCDR Community Affairs Domain IRT score	0.98 (0.97-1.00)	<.0001	0.97 (0.95 - 1.00)
	eCDR Community Affairs Domain IRT score + age	0.99 (0.97-1.00)	<.0001	0.98 (0.97 - 1.00)
	eCDR Community Affairs Domain IRT score + gender	0.99 (0.98-1.00)	<.0001	0.99 (0.97 - 1.00)
	eCDR Community Affairs Domain IRT score + education	0.99 (0.97-1.00)	<.0001	0.99 (0.97 - 1.00)
	eCDR Community Affairs Domain IRT score + age + gender + education	1.00	<.0001	0.96 (0.93 - 1.00)
	age + gender + education	0.87 (0.74-1.00)	<.0001	0.85 (0.80 - 0.90)
CDR Orientation Domain box score	eCDR Orientation Domain IRT score	0.86 (0.72-1.00)	<.0001	0.85 (0.81 - 0.89)
	eCDR Orientation Domain IRT score + age	0.86 (0.72-1.00)	<.0001	0.84 (0.80 - 0.87)
	eCDR Orientation Domain IRT score + gender	0.90 (0.80-0.99)	<.0001	0.85 (0.81 - 0.88)
	eCDR Orientation Domain IRT score + education	0.91 (0.83-0.99)	<.0001	0.84 (0.80 - 0.88)
	eCDR Orientation Domain IRT score + age + gender + education	0.91 (0.82-0.99)	<.0001	0.84 (0.80 - 0.88)
	age + gender + education	0.81 (0.69-0.93)	<.0001	0.76 (0.71 - 0.81)
CDR Memory Domain box score	eCDR Memory Domain IRT score	0.79 (0.71-0.87)	<.0001	0.79 (0.72 - 0.87)
	eCDR Memory Domain IRT score + age	0.79 (0.71-0.88)	<.0001	0.79 (0.72 - 0.86)
	eCDR Memory Domain IRT score + gender	0.79 (0.71-0.88)	<.0001	0.79 (0.71 - 0.86)
	eCDR Memory Domain IRT score + education	0.80 (0.72-0.88)	<.0001	0.79 (0.72 - 0.85)
	eCDR Memory Domain IRT score + age + gender + education	0.80 (0.72-0.88)	<.0001	0.79 (0.71 - 0.86)
	age + gender + education	0.63 (0.54-0.71)	0.0037	0.58 (0.49 - 0.66)
CDR Judgment and Problem Domain box score	eCDR Judgment and Problem Solving Domain IRT score	0.76 (0.59-0.93)	0.0024	0.76 (0.60 - 0.93)
	eCDR Judgment and Problem Solving Domain IRT score + age	0.78 (0.61-0.95)	0.0009	0.78 (0.63 - 0.92)
	eCDR Judgment and Problem Solving Domain IRT score + gender	0.80 (0.65-0.95)	<.0001	0.80 (0.67 - 0.93)
	eCDR Judgment and Problem Solving Domain IRT score + education	0.77 (0.62-0.93)	0.0004	0.77 (0.62 - 0.91)
	eCDR Judgment and Problem Solving Domain IRT score + age + gender + education	0.82 (0.69-0.95)	<.0001	0.79 (0.66 - 0.92)
	age + gender + education	0.58 (0.43-0.72)	0.3056	0.51 (0.36 - 0.66)

© 2023 Nosheny RL et al. *JAMA Network Open.*

CDR global	eCDR IRT global score	0.79 (0.70-0.87)	<.0001	0.78 (0.70 - 0.86)
	eCDR IRT global score + age	0.79 (0.70-0.87)	<.0001	0.78 (0.71 - 0.85)
	eCDR IRT global score + gender	0.79 (0.71-0.88)	<.0001	0.79 (0.71 - 0.86)
	eCDR IRT global score + education	0.80 (0.72-0.88)	<.0001	0.78 (0.71 - 0.85)
	eCDR IRT global score + age + gender + education	0.80 (0.72-0.88)	<.0001	0.78 (0.71 - 0.85)
	age + gender + education	0.63 (0.54-0.71)	0.0037	0.58 (0.49 - 0.66)

CDR box score or global score was used as a binary outcome (0, ≥0.5).

*** For Personal Care domain, there are only 3 participants with domain box score** ≥0.5, which are not enough to perform the10-fold cross-validation and therefore cvAUCs are not provided.

© 2023 Nosheny RL et al. *JAMA Network Open.*

eFigure: Heat Map of Correlations Between CDR or eCDR and UDS Neuropsychological Assessments

**UDS Assessment**	CDR sum of box	eCDR IRT global score	Community affairs domain IRT score	Judgment and problem solving domain IRT score	Memory domain IRT score	Orientation domain IRT score	Personal care domain IRT score
Digit span forwards	0.024	0.051	0.037	0.066	0.051	0.051	0.039
Digit span backwards	0.063	0.083	0.044	0.082	0.089	0.101	0.041
Benson	0.093	0.068	0.036	0.062	0.063	0.086	0.069
Trail Making test A	0.105	0.172	0.138	0.206	0.173	0.155	0.155
Semantic fluency	0.127	0.245	0.216	0.248	0.238	0.243	0.227
MINT naming test	0.128	0.050	0.051	0.055	0.050	0.049	0.044
Benson Delayed	0.173	0.158	0.132	0.168	0.155	0.159	0.116
Trail Making Test B	0.224	0.276	0.233	0.280	0.280	0.276	0.246
CRAFT Learning	0.237	0.229	0.187	0.198	0.238	0.242	0.207
CRAFT Memory delayed	0.286	0.254	0.206	0.217	0.265	0.272	0.210
MOCA total score	0.295	0.349	0.305	0.354	0.352	0.339	0.324
FAQ	0.371	0.344	0.340	0.352	0.340	0.324	0.353

Cells are color-coded according to the magnitude of the Spearman correlation. Deeper color represents higher correlation. Correlations were not significantly different between (1) eCDR IRT score and UDS assessment; (2) CDR-SB and UDS assessment. (See **eTable 4).**

© 2023 Nosheny RL et al. *JAMA Network Open.*

eTable 5: Difference in Correlations With Uniform Data Set (UDS) Assessments Between eCDR IRT Score and CDR-Sum of Boxes.

UDS Measure	p-value
Digit span forwards	0.31
Digit span backwards	0.79
Benson	0.73
Trail Making test A	0.37
Semantic fluency	0.10
MINT naming test	0.29
Benson Delayed	0.84
Trail Making Test B	0.47
CRAFT Learning	0.91
CRAFT Memory delayed	0.64
MOCA total score	0.44
FAQ_score	0.69

UDS=Uniform Data Set; MINT=Multilingual Naming Test; MOCA=Montreal Cognitive Assessment; FAQ=Functional Activities Questionnaire. P-values compare the difference in correlations between UDS assessments indicated and (1) eCDR IRT score; (2) CDR-Sum of Box scores.

eReferences

1. Weiner MW, Aaronson A, Eichenbaum J, et al. Brain health registry updates: An online longitudinal neuroscience platform. *Alzheimer's & dementia : the journal of the Alzheimer's Association*. Mar 25 2023;doi:10.1002/alz.13077

2. Nosheny RL, Camacho MR, Insel PS, et al. Online study partner-reported cognitive decline in the Brain Health Registry. *Alzheimers Dement (N Y)*. 2018;4:565-574. doi:10.1016/j.trci.2018.09.008

3. Li Y, Xiong C, Aschenbrenner AJ, et al. Item response theory analysis of the Clinical Dementia Rating. *Alzheimer's & dementia : the journal of the Alzheimer's Association*. Mar 2021;17(3):534-542. doi:10.1002/alz.12210

© 2023 Nosheny RL et al. *JAMA Network Open.*

4. Liu Y, Li Z, Liu H. Reporting Valid and Reliable Overall Scores and Domain Scores Using Bi-Factor Model. *Appl Psychol Meas*. Oct 2019;43(7):562-576. doi:10.1177/0146621618813093

5. Weintraub S, Besser L, Dodge HH, et al. Version 3 of the Alzheimer Disease Centers' Neuropsychological Test Battery in the Uniform Data Set (UDS). *Alzheimer disease and associated disorders*. Jan-Mar 2018;32(1):10-17. doi:10.1097/wad.0000000000000223

6. Welsh KA, Butters N, Mohs RC, et al. The Consortium to Establish a Registry for Alzheimer's Disease (CERAD). Part V. A normative study of the neuropsychological battery. *Neurology*. Apr 1994;44(4):609-14.

7. Gaudino EA GM, Squires NK. Construct validity in the Trail Making Test: what makes Part B harder? *J Clin Exp Neuropsychol*. 1995;17(4):529-535.

8. Kaufer DI, Cummings JL, Ketchel P, et al. Validation of the NPI-Q, a brief clinical form of the Neuropsychiatric Inventory. *The Journal of neuropsychiatry and clinical neurosciences*. Spring 2000;12(2):233-9.

9. Yesavage JA, Brink TL, Rose TL, et al. Development and validation of a geriatric depression screening scale: a preliminary report. *Journal of psychiatric research*. 1982;17(1):37-49.

10. Nasreddine ZS, Phillips NA, Bedirian V, et al. The Montreal Cognitive Assessment, MoCA: a brief screening tool for mild cognitive impairment. *Journal of the American Geriatrics Society*. Apr 2005;53(4):695-9. doi:10.1111/j.1532-5415.2005.53221.x

11. Kaplan E GH, Weintraub S. The Boston Naming Test. *Philadelphia, PA Lea and Febiger*. 1983;

12. Beekly DL, Ramos EM, Lee WW, et al. The National Alzheimer's Coordinating Center (NACC) database: the Uniform Data Set. *Alzheimer disease and associated disorders*. Jul-Sep 2007;21(3):249-58. doi:10.1097/WAD.0b013e318142774e

13. Weintraub S, Salmon D, Mercaldo N, et al. The Alzheimer's Disease Centers' Uniform Data Set (UDS): the neuropsychologic test battery. *Alzheimer disease and associated disorders*. Apr-Jun 2009;23(2):91-101. doi:10.1097/WAD.0b013e318191c7dd

14. Ivanova I, Salmon DP, Gollan TH. The multilingual naming test in Alzheimer's disease: clues to the origin of naming impairments. *Journal of the International Neuropsychological Society : JINS*. Mar 2013;19(3):272-83. doi:10.1017/S1355617712001282

© 2023 Nosheny RL et al. *JAMA Network Open.*
