# Supplementary material for: Evaluation of the Electronic Clinical Dementia Rating for Dementia Screening
Source: JAMA Netw Open. 2023 Sep 14;6(9):e2333786. doi: 10.1001/jamanetworkopen.2023.33786 (PMC10502518; doi:10.1001/jamanetworkopen.2023.33786)
Supplement: Supplement 2. — Data Sharing Statement [file jamanetwopen-e2333786-s002.pdf]

Data Sharing Statement

Nosheny. Evaluation of the Electronic Clinical Dementia Rating for Dementia Screening. *JAMA Netw Open*. Published September 14, 2023. doi:10.1001/jamanetworkopen.2023.33786

Data
Data available: No

Additional Information
Explanation for why data not available: The data will not be shared due to data sharing policies of the study sites.
